# The combined effects of simulated microgravity and X-ray radiation on MC3T3-E1 cells and rat femurs

**DOI:** 10.1038/s41526-021-00131-1

**Published:** 2021-02-15

**Authors:** Jingjing Dong, Honghui Wang, Gaozhi Li, Ke Wang, Yingjun Tan, Lijun Zhang, Yixuan Wang, Zebing Hu, Xinsheng Cao, Fei Shi, Shu Zhang

**Affiliations:** 1 The Key Laboratory of Aerospace Medicine, Ministry of Education, Air Force Medical University, Xi’an, Shaanxi China; 2 Rehabilitation Physiotherapy Department, Lintong Rehabilitation and Recuperation Center, PLA Joint Logistic Support Force, Xi’an, Shaanxi China; 3 State Key Laboratory of Space Medicine Fundamentals and Application, China Astronaut Research and Training Center, Beijing, China

**Keywords:** Biochemistry, Cell biology

## Abstract

Microgravity is well-known to induce Osteopenia. However, the combined effects of microgravity and radiation that commonly exist in space have not been broadly elucidated. This research investigates the combined effects on MC3T3-E1 cells and rat femurs. In MC3T3-E1 cells, simulated microgravity and X-ray radiation, alone or combination, show decreased cell activity, increased apoptosis rates by flow cytometric analysis, and decreased Runx2 and increased Caspase-3 mRNA and protein expressions. In rat femurs, simulated microgravity and X-ray radiation, alone or combination, show increased bone loss by micro-CT test and Masson staining, decreased serum BALP levels and Runx2 mRNA expressions, and increased serum CTX-1 levels and Caspase-3 mRNA expressions. The strongest effect is observed in the combined group in MC3T3-E1 cells and rat femurs. These findings suggest that the combination of microgravity and radiation exacerbates the effects of either treatment alone on MC3T3-E1 cells and rat femurs.

## Introduction

Space programs have been clearly shifted toward long-term space missions^1–3^. Severe bone loss in astronauts is still an important medical concern. Microgravity (MG) and space radiation (RA), as the major space–environment factors, have shown various effects on the human body, especially on bone tissue^4–6^. Studies have demonstrated that the balance of bone tissue depends on the balance of osteogenesis and bone resorption, which is mediated by osteoblasts and osteoclasts^7,8^. The conditions in space cause an imbalance between bone formation and resorption, which results in bone loss^5^. Many studies, including cell studies, animal studies, and bedrest research, have demonstrated that MG results in osteopenia^9–11^. Genomic analysis revealed that the expression of hundreds of genes in osteoblasts is altered by MG^12,13^. In the lab, 1 Gy protons^14^ or 2 Gy X-rays^15^ could simulate the space radiation that causes a significant and long-term loss of trabecular microstructure. Further studies showed that RA decreases the number of active osteoblasts, decreases osteogenesis, and increases bone loss^16,17^. However, many studies have been designed to investigate clinical radiotherapy in tumors^18^. These studies indicated that RA causes direct damage to bone tissue, causing growth disturbances, osteopenia, and incomplete fracture healing^19^. Although there are many studies on the bone loss induced by MG or RA, the actual phenomenon and mechanism of the combined effects of these two factors are still not clear, and relevant research is extremely limited. The research on the mechanism of the bone loss induced by MG focuses majorly on alterations in protein signal transduction^7,20^, while the biological damage caused by the RA is one of the research hotspots, such as DNA strand breaks or membrane damage^21^. Even though the contents and the directions of the study are various, the combination studies of MG and the RA are rare. Therefore, it is necessary to furtherly examine the combined effects to obtain deep insight into the effects of space and to develop effective preventative measures.

## Results

### The effects of clinorotation and X-ray RA on the activity of MC3T3-E1 cells

**Fig. 1:**
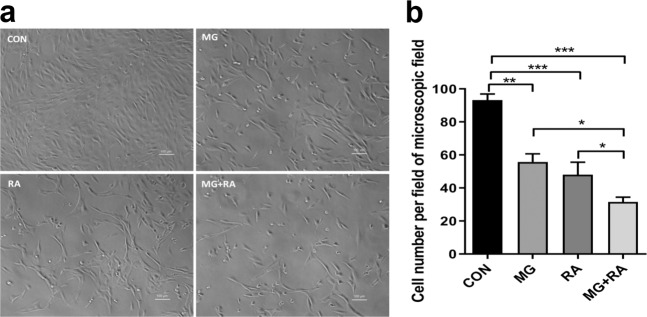
The combination of clinorotation and X-ray radiation decreased the survival of MC3T3-E1 cells as evidenced by cell counting results. **a** Representative images of MC3T3-E1 cells in the four groups (scale bars, 100 µm). **b** The number of surviving cells in the four groups (*n* = 4). The data are expressed as the mean ± SD. *P < 0.05, **P < 0.01, ***P < 0.001, ns not significant.

In the CON group, long fusiform MC3T3-E1 cells densely covered the flat bottom of the well, notably overlapping. In the other three groups, in contrast to the CON group, the picture showed sparse cell coverage, without overlap. The MG + RA group showed the least cell coverage among the groups (Fig. 1a). As shown in Fig. 1b, the number of surviving MC3T3-E1 cells in the four groups was 93.25 ± 7.41 × 10^3^, 55.75 ± 9.98 × 10^3^, 48.0 ± 15.29 × 10^3^, and 31.5 ± 5.97 × 10^3^. The number of surviving cells in the treated groups was reduced compared with the CON group, and the MG + RA group (31.5 ± 5.97 × 10^3^) showed the largest reduction among the groups.

### Clinorotation and X-ray RA inhibited osteoblast differentiation and increased osteoblast apoptosis in MC3T3-E1 cells

**Fig. 2:**
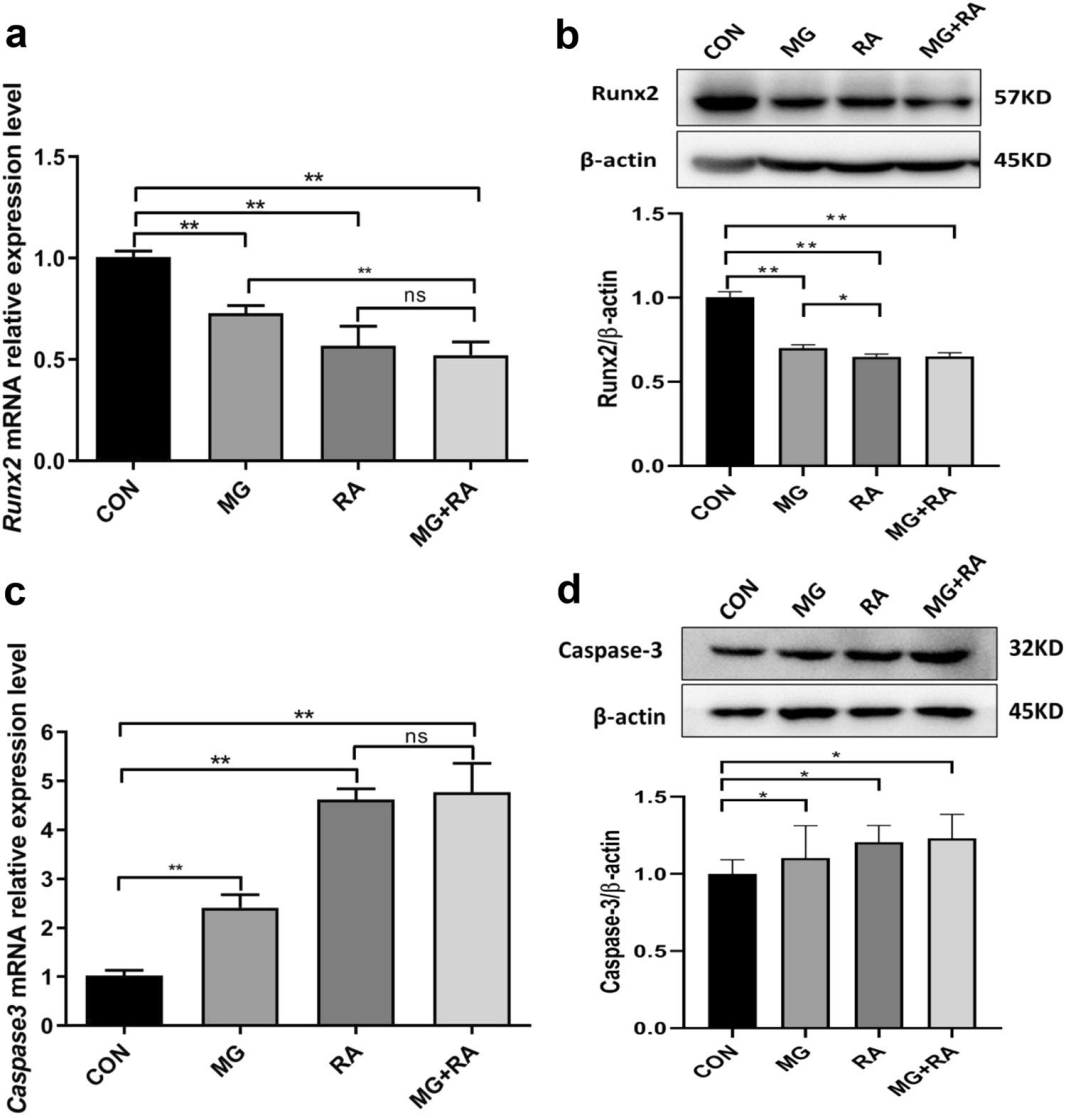
Clinorotation and X-ray radiation inhibited osteoblast differentiation and increased osteoblast apoptosis in MC3T3-E1 cells as evidenced by qRT-PCR and Western blot analysis. **a**, **c** qRT-PCR analysis of osteoblast marker genes (Runx2 and Caspase-3) in the four groups of MC3T3-E1 cells (*n* = 3). **b**, **d** Western blot analyses of osteoblast marker genes (Runx2 and Caspase-3) in the four groups of MC3T3-E1 cells (*n* = 3). The data are expressed as the mean ± SD. *P < 0.05, **P < 0.01, ***P < 0.001, ns not significant. All blots derived from the same experiment and were processed in parallel.

**Fig. 3:**
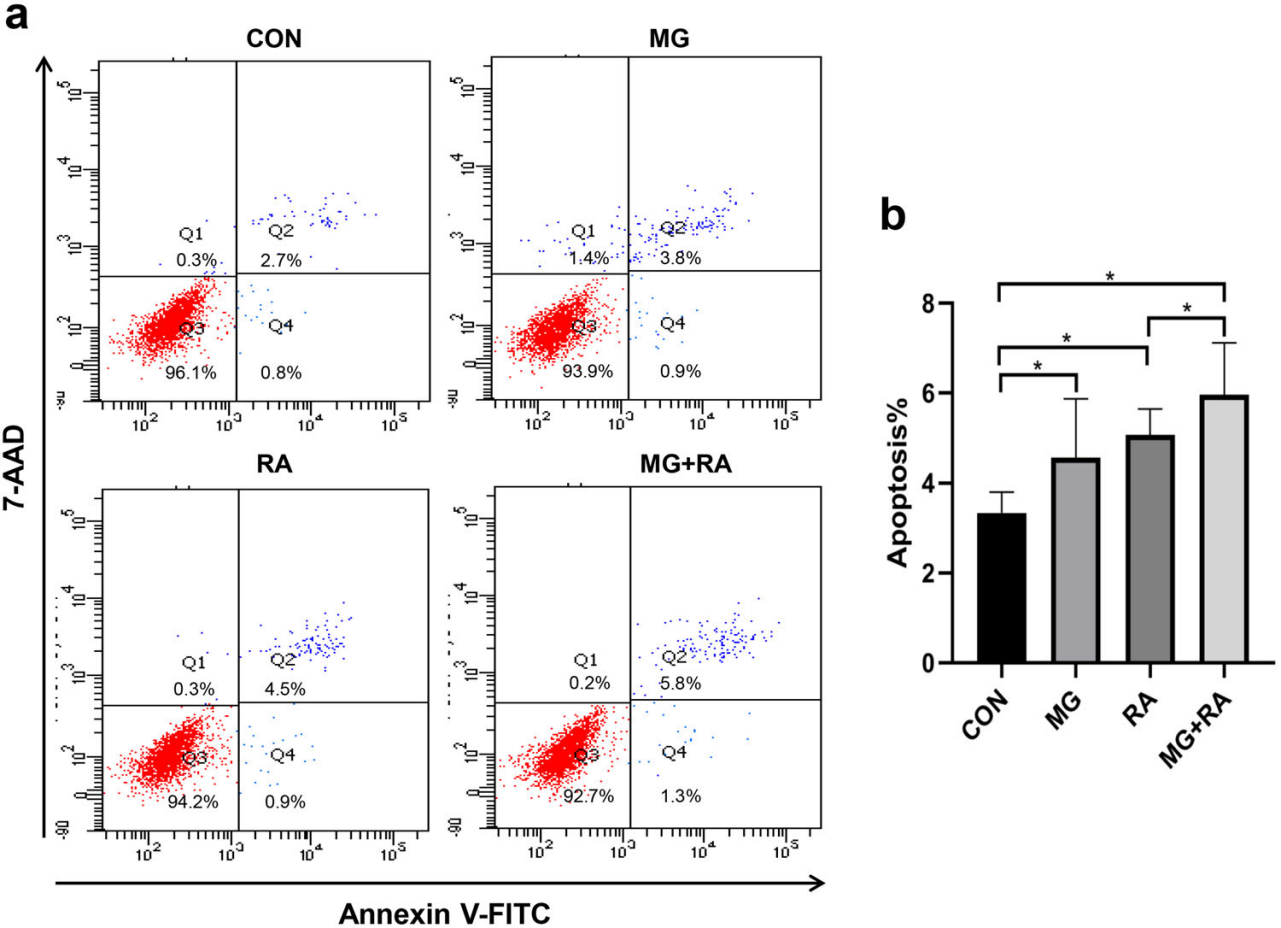
Clinorotation and X-ray radiation increased osteoblast apoptosis in MC3T3-E1 cells as evidenced by Flow cytometry. **a** Flow cytometric analysis of apoptosis in osteoblasts stained with Annexin V-FITC. **b** Apoptosis rates of four groups in MC3T3-E1 cells (*n* = 3) which were reflected by the sum of Q2 and Q4 quadrant. The data are expressed as the mean ± SD. *P < 0.05, **P < 0.01, ***P < 0.001, ns not significant.

To investigate the mechanism of the different cell activities in the four groups, Runx2 (runt-related transcription factor 2, the differentiation marker of osteoblast) and Caspase-3 (the apoptosis marker of osteoblast) were chosen to partly evaluate the ability of osteoblast differentiation and osteoblast apoptosis (Fig. 2). After treatment clinorotation for 48 h or with 2 Gy X-ray RA, the mRNA expression and intracellular protein expression of Runx2 were decreased compared to the CON group. Among the groups, the mRNA expression and intracellular protein expression of Runx2 decreased the most in the MG + RA group, which showed the lowest expression. Conversely, the mRNA expression and intracellular protein expression of Caspase-3 in the three treated groups increased compared with that of the CON group, and the expression level of the MG + RA group increased the most among the four groups. The apoptosis rate of MC3T3-E1 cells by flow cytometry analysis also supported that RA-treated or MG-treated increased osteoblast apoptosis and MG + RA-treated increased most in the four groups (Fig. 3).

### The effects of hindlimb unloading and X-ray RA on rat femurs

**Fig. 4:**
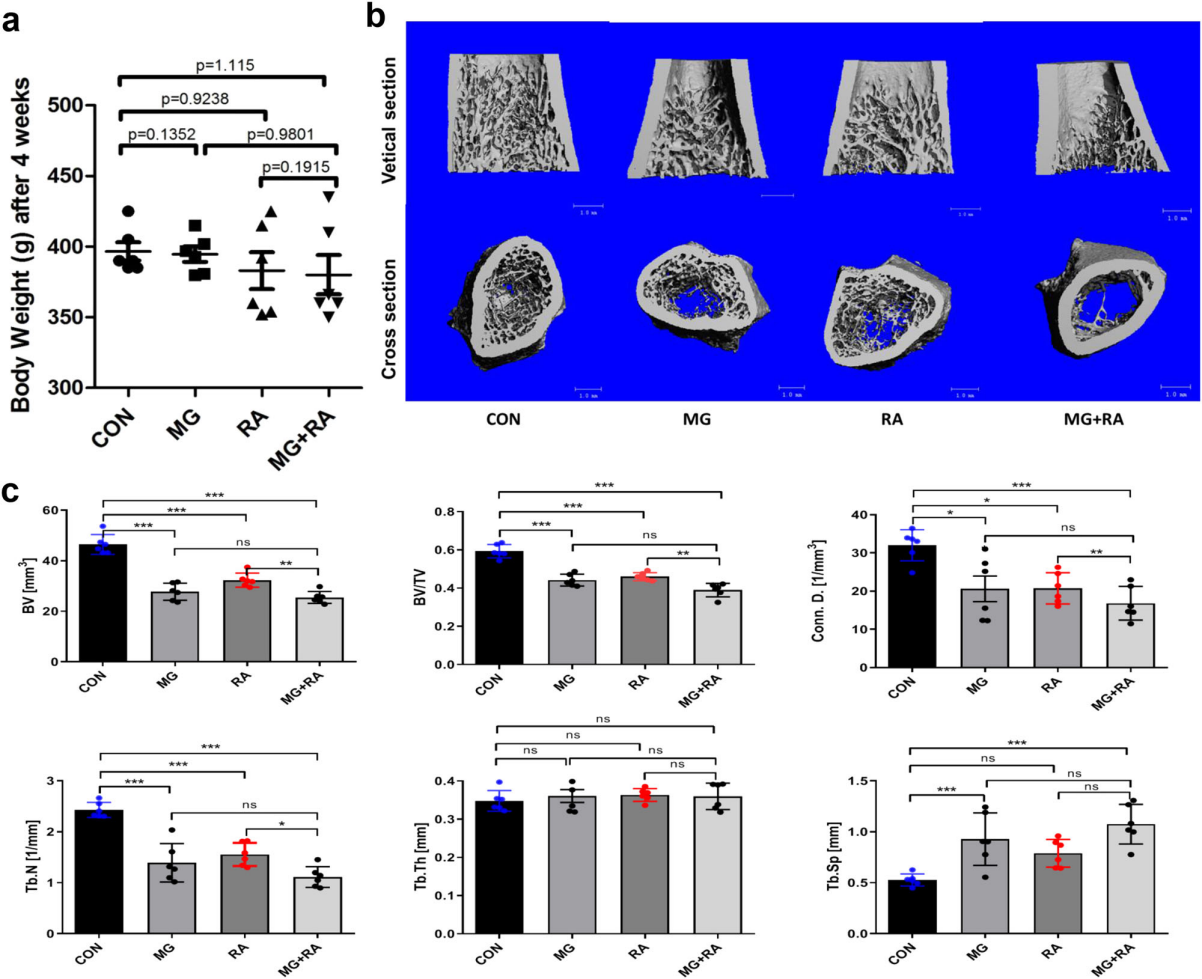
Hindlimb unloading and X-ray radiation aggravate osteopenia development as evidenced by the bone morphology of distal femurs evaluated by micro-CT analysis. **a** Rat body weights of four groups after 4 weeks (*n* = 6). **b** 3D micro-CT images within the distal femur of the four groups. **c** The three-dimensional architecture parameters of micro-CT analysis of four groups (*n* = 6). The data are expressed as the mean ± SD. *P < 0.05, **P < 0.01, ***P < 0.001, ns not significant.

Rat body weights of the four groups have nonsignificant differences suggested that the stress caused by the tail suspension or RA was well tolerated by rats (Fig. 4a). As evidence by micro-CT, 3D image reconstruction of rat distal femurs showed that among the groups, the CON group showed the most complete trabecular structures and the largest bone mass. The other three groups showed more or less sparse, fractured and discontinuous trabecular architecture, and the trabecular structures and bone mass in the MG + RA group were the most severely damaged (Fig. 4b). The 3D architecture parameters also showed similar corresponding changes, with significant decreases in BV, BV/TV, Conn. D, and Tb. N, no significance in Tb. Th, and increases in Tb. Sp by treatment with hindlimb unloading or X-ray RA alone or together. The MG + RA group displayed the lowest BV (25.49 ± 2.39 mm^3^), the lowest BV/TV (0.39 ± 0.04), the lowest Conn. D (16.83 ± 4.41 1/mm^3^), the lowest Tb. N (1.113 ± 0.203 1/mm), and the highest Tb. Sp (1.075 ± 0.19 mm) among the four groups (Fig. 4c).

**Fig. 5:**
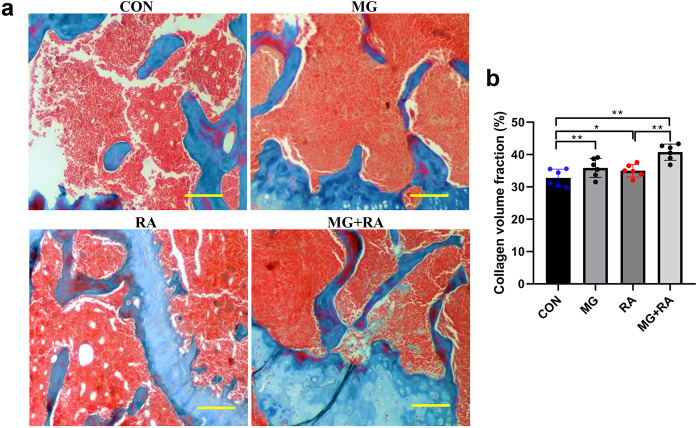
Hindlimb unloading and X-ray radiation aggravated osteopenia development in rat femurs as evidenced by Masson staining. **a** Representative Masson staining images of rat distal femurs in the four groups and the blue part indicates collagen (scale bars, 50 µm). **b** Collagen volume fraction of four groups in rat femurs (*n* = 6). The data are expressed as the mean ± SD. *P < 0.05, **P < 0.01, ***P < 0.001, ns not significant.

Masson staining further confirmed that hindlimb unloading and X-ray RA aggravated osteopenia development as evidenced by collagen volume fraction (the ratio of collagen area to total area). As shown in Fig. 5, the results showed that in the CON group the blue-stained collagen was in good shape and uniformly stained, and collagen volume fraction was the lowest. In the other three groups, the collagen showed different degrees of deformation and uneven staining, and collagen volume fraction increased. The MG + RA group showed the most obvious changes.

### Hindlimb unloading and X-ray RA inhibited osteogenesis and promoted bone absorption in rat femurs

**Fig. 6:**
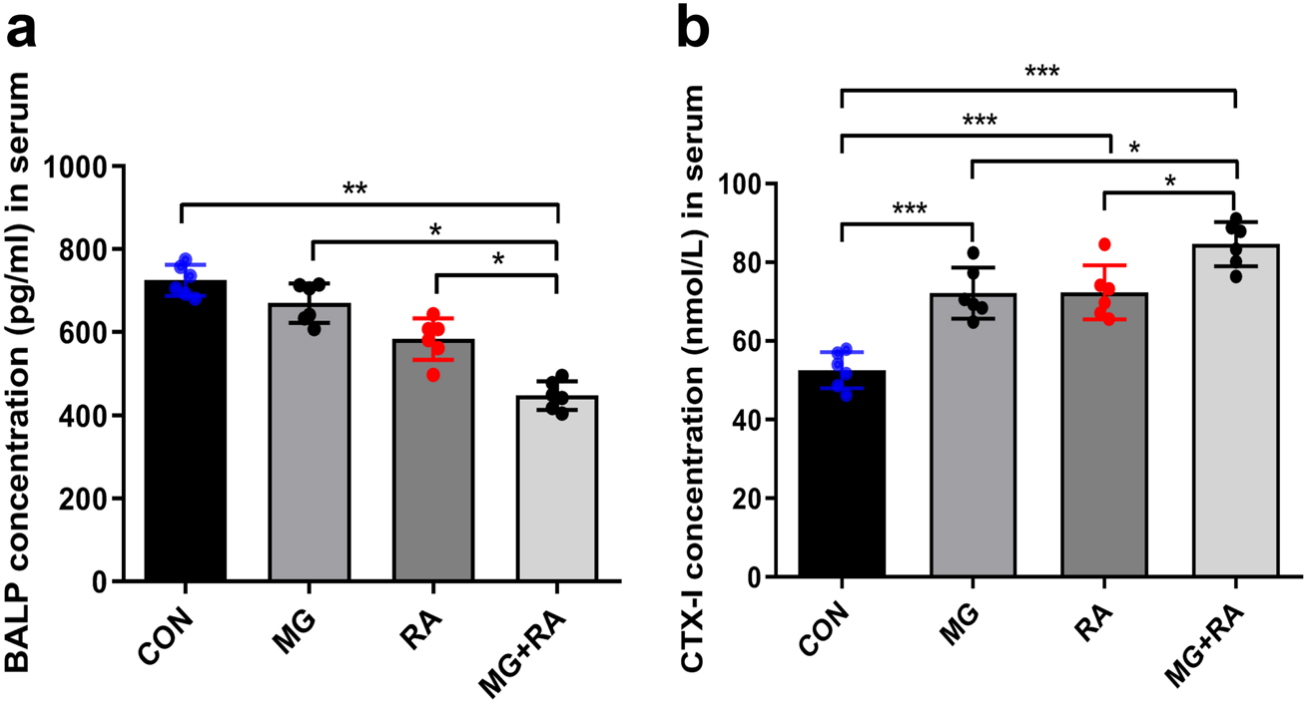
Hindlimb unloading and X-ray radiation inhibited osteogenesis and increased bone absorption in rats as evidenced by serum analysis. **a**, **b** Serum analysis of BALP and CTX-1 in the four groups of rats (*n* = 6). The data are expressed as the mean ± SD. *P < 0.05, **P < 0.01, ***P < 0.001, ns not significant.

Compared with the CON group, the other three treated groups exhibited decreased expression levels of the osteogenesis marker BALP (bone-specific alkaline phosphatase) and increased expression levels of the bone absorption marker CTX-1 (a carboxy-terminal peptide of type I collagen). The MG + RA group showed the most dramatic changes among the groups, the lowest level of BALP (186.0 ± 123.0 pg/ml) and the highest level of CTX-1 (78.9 ± 8.6 nmol/L) (Fig. 6).

**Fig. 7:**
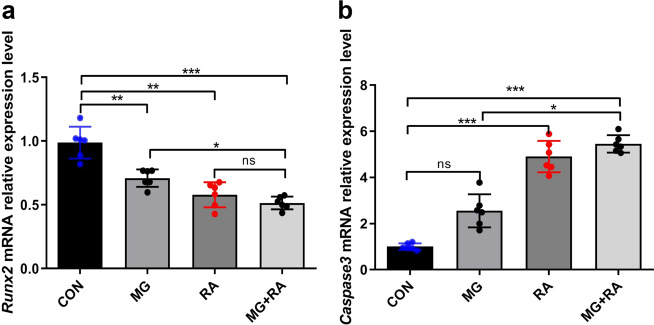
Hindlimb unloading and X-ray radiation inhibited osteoblast differentiation and increased osteoblast apoptosis in rat femurs as evidenced by qRT-PCR analysis. **a**, **b** qRT-PCR analysis of Runx2 and Caspase-3 in the four groups of rat femurs (*n* = 6). The data are expressed as the mean ± SD. *P < 0.05, **P < 0.01, ***P < 0.001, ns not significant.

In addition, Runx2 and Caspase-3 contribute to osteoblast differentiation and apoptosis in rat femurs (Fig. 7). After treatment with hindlimb unloading or X-ray RA alone or together, the mRNA expression of Runx2 decreased compared to that in the CON group, and Runx2 (0.56 ± 0.12) decreased the most in the MG + RA group. Conversely, the mRNA expression of Caspase-3 increased in the three treatment groups compared with the CON group, and the expression level (5.44 ± 0.57) in the MG + RA group was the highest.

## Discussion

Until now, the combined effect and mechanism of MG and RA on bone have not been elucidated. The present study demonstrates that the combination of MG and X-ray RA has a synergistic effect on the activity of MC3T3-E1 cells and on bone loss in rat femurs. Moreover, these findings also revealed that the combined treatment inhibited differentiation and increased apoptosis in MC3T3-E1 cells, and inhibited bone formation and increased bone resorption in rat femurs.

MG and RA, as the two most important factors during space flight missions, have been shown to have various influences on human health. Space RA mainly consists of a complex mix of ions from solar particle events (SPEs) and galactic cosmic radiation (GCR)^22^. During extended missions in space, estimated tissue dose rates from GCR would be approximately 0.4–0.8 mGy/d and 1–2.5 mSv/d. SPE dose rates may reach as high as 50 mGy/h inside a shielded vehicle and approximately 250 mGy/h exposed during extravehicular activity in deep space^23^. Given SPEs occur randomly and can deliver a relatively high dose (up to 2 Gy) over a short period of time, and the duration of future missions planned by NASA to nearby asteroids and Mars, space radiation could deliver a whole-body radiation dose of approximately 2 Gy^24^. These RAs are large in low linear energy transfer, and therefore their biological effects are similar to gamma and X-ray. Some laboratories have studied the effect of space RA on long-term bone loss following a dose of 1 Gy^14^ proton or 2 Gy X-rays^15^ to simulate these potential conditions. To truly simulate the MG conditions, two kinds of models are commonly chosen: the cell clinorotation model and the hindlimb unloading rat model^7,20^. A few combination studies used MG following RA^25,26^, as osteopenia occurred rapidly in the animal models and continued a long-term suppression of bone formation was observed after exposure^14,26^. Both the cell model and the animal model are mature models widely used in biological researches, therefore, the results of these models have important reference value for human research. Therefore, in this study the combined model of 2 Gy X-ray RA followed by clinorotation or hindlimb unloading to simulate space MG as realistically as possible.

MC3T3-E1 cells, as the target cell type in this study, are a kind of pre-osteoblast. As the pre-osteoblastic MC3T3-E1 cell has strong abilities of proliferation, mineralization, and differentiation at the early stage of differentiation, it has been widely used to study the biological alterations of osteoblasts in vitro^20^. Damage to osteoblasts within the bone microenvironment is thought to be a primary contributor to reducing bone mass causing osteopenia^27,28^. In this study, the changes of the differentiation marker Runx2 and the apoptosis marker Caspase-3 demonstrated that MG and RA could inhibit the activity of MC3T3-E1 cells by inhibiting differentiation and increasing apoptosis, and the data of the flow cytometry also support the increased apoptosis activity. In many cell-based studies and our previous studies, exposure to rotating wall vessel or random positioning machine systems to simulate MG in the laboratory significantly inhibited differentiation and mineralization while increasing apoptosis of osteoblasts^7,29,30^. RA induced decreased osteoblast proliferation and differentiation, cell cycle arrest, reduced collagen production, and increased sensitivity to apoptotic agents^16,31–33^. Limited osteoblast cell research has evaluated the combined effect. X-ray RA exacerbated the effects of MG on bone density loss, cell proliferation, and mineralization decreased combined high-frequency acoustic wave signals and increased combined low-intensity pulsed ultrasound in a simulated MG environment^33,34^. In this study, in MC3T3-E1 cells, the combination of RA and MG also exacerbated the decreased osteoblast differentiation and increased osteoblast apoptosis, induced by the individual treatments, and the exact underlying mechanism requires further research.

This in vivo study revealed that the combined MG and RA inhibited bone formation and increased bone resorption in rat femurs. The structural parameters of BV, BV/TV, Conn. D, Tb. Th, Tb. N and Tb. Sp in micro-CT test reflects the balance of bone formation and bone resorption. Micro-CT analysis and Masson staining demonstrated that simulated MG or RA could induce bone loss, and the combined treatment deteriorated the loss of bone. This detrimental effect may decrease the maintenance ability of bone biomechanical property and structural integrity, leading to increased susceptibility to fracture. BALP and CTX-1, two bone metabolism markers in the serum, were chosen to be tested. BALP is a bone formation marker, it can regulate the hydrolysis of phosphate and pyrophosphate to promote bone formation; CTX-1 is a bone resorption marker, which specifically reflects the decomposition of type I collagen in bone tissue. The changing of BALP and CTX-1 in the serum showed a more decreased bone formation and increased bone resorption after the combined treatment compared with the single treatment. Runx2, which is frequently described as the master gene of osteoblast differentiation, decreased mRNA expression level in the femur. And Caspase-3, which is a master regulator of cell apoptosis, increased mRNA expression level in the femur. These data further support the result of decreased bone formation illustrating by the change of BALP in the rat serum^7,35^. Even though the functions of osteoclast are not tested in this study, the increased activity of osteoclast could be inferred from the increased concentration of CTX-1 in the serum. Therefore, the changes of osteoclast function under combined conditions of simulated MG and X-ray RA may need to be clarified in further study. Some scientists have identified a reduction in bone strength after exposure with time and at various locations within the bone. The clinically relevant, high dose exposures caused relatively greater damage to spongy trabecular bone compared to dense cortical bone^14^, and heavy-ion RA at relatively low doses of 50 cGy could cause an increase in cortical bone porosity^36^. However, some studies found that spaceflight-relevant doses did not change the body mass, activity level, or nutritional status of mice^15,37,38^. The hindlimb unloading (HU) model used to simulate MG in the laboratory successfully presented osteopenia characterized by decreased bone mineral content, weakened bone resistance, and femoral bone loss^39,40^. Studies of the combined effect of spaceflight-relevant types and doses of RA and MG on bone have not received much attention. Shane A. Lloyd et al established a combined mouse model and found an additive effect on trabecular and cortical bone^26^. Combined RA and MG resulted in more severe bone loss, and the specific mechanism needs to be further studied.

In conclusion, the combined effects of MG and X-ray RA in MC3T3-E1 cells and rat femurs had a synergistic effect to deteriorate the adverse effect. Further studies are required to investigate the exact underlying mechanism, to find a potential therapeutic target of bone loss induced by the combined effect of MG and RA in space.

## Materials and methods

### Cell culture

MC3T3-E1 cells, the pre-osteoblast cells as the research object, purchased from the Cell Bank of the Chinese Academy of Sciences (Beijing, China). Cells were cultured in a mixed solution of α-MEM containing 10% fetal bovine serum (HyClone, USA) and 1% penicillin/streptomycin (HyClone, USA) in an incubator at 37 °C with 5% CO_2_. The medium was changed after 2 days. When the cells reached 90% confluence, they were subcultured after digestion with trypsin (Millipore, USA) for the experiments. Cells were randomly grouped into four groups: the MG group, which was rotated in a 2D clinorotation for 48 h; the RA group, which was exposed to 2 Gy X-ray and cultured for 48 h; the combined (MG + RA) group, which was first exposed to 2 Gy X-ray followed by rotation in a 2D clinorotation for 48 h; and the control (CON) group, which was cultured for 48 h without any treatment.

### Animals

Seven-week-old male Sprague–Dawley (SD) rats were chosen for this study. Animals were randomly assigned to four groups (*n* = 6): the MG group, administered HU for 4 w; the RA group, exposed to 2 Gy X-ray and then maintained for 4 w; the combined (MG + RA) group, exposed to 2 Gy X-ray and then hindlimb unloading for 4 w; and the control (CON) group, which was maintained for 4 w without any treatment.

All rats were maintained in accordance with the guidelines of the Committees of Animal Ethics and Experimental Safety of the Air Force Medical University. All experimental protocols were approved by the Animal Care Committee of Air Force Medical University.

### Clinorotation to simulate MG

In this experiment, a two-dimensional clinostat designed by the China Astronaut Research and Training Center was used to simulate the effects of MG. MC3T3-E1 cells were incubated at 37 °C in 25 cm^2^ cell culture flasks (Corning Incorporated, USA) or plated on 25-mm glass coverslips and filled with culture medium. To avoid the influence of shear stress, all culture flasks were filled with medium to exclude air bubbles and hermetically closed during rotation. The cells were rotated around a horizontal axis with centrifugation at 1.29 × 10^−2^ × *g*, which resulted in the randomization of the gravitational vector. That was equivalent to the MG of low earth orbit (about 0.01*g*). The 2D clinostat has been described previously^41^.

### Hindlimb unloading rat model

Rats were suspended by the tail individually, using a strip of adhesive surgical tape attached to a chain hanging on a pulley, and maintained at an angle of 30° with only the forelimbs on the floor for 4 w. The suspended rats could move freely.

### Radiation

Rats were anesthetized and irradiated in the prone position with a single field of 180 kV X-rays to a dose of 2 Gy at a rate of 2 Gy/min (MultiRad225; Faxitron, USA). Cells were placed in the vessel and then irradiated at a dose of 2 Gy X-rays.

### Image-based cultured cell counting

The MC3T3-E1 cells were cultured on slides in 6-well plates with a density of 1.2 × 10^6^ cells/ml for 24 h. Then the slides in the RA and MG + RA group were irradiated at a dose of 2 Gy X-rays. After irradiation, the slides in the MG and MG + RA groups were treated by clinorotation for 48 h, the CON and RA groups were also cultured for 48 h under the same culture conditions. The slides were collected and washed gently with PBS and fixed with 4% paraformaldehyde solution for 30 min. Images of cells in every slide were acquired concurrently with phase contrast and fluorescence microscopy using a Nikon TE300 microscope with a ×10 objective. The average number of cells per four visual fields was counted with the included software.

### qRT-PCR

Total RNA was extracted from MC3T3-E1 cells (Invitrogen-, USA) or rat femurs (Invitrogen-, USA). cDNA was prepared by a PrimeScript^®^ RT reagent kit (TakaRa code: DRR037, Japan). According to conventional protocols^42^, the target genes were quantified by an ABI 7500 real-time PCR system (Applied Biosystems) using SYBR^®^ Premix Ex TaqTM II (TaKaRa code: DRR820A). The primer pairs were as follows: Runx2: F-5′-CCA TAA CGG TCT TCA CAA ATC C-3′ and R-5′-GCG GGA CAC CTA CTC TCA TAC T-3′; Caspase-3: F-5′-GAG CTT GGA ACG GTA CGC TA -3′ and R-5′-CCG TAC CAG AGC GAG ATG AC-3′; GAPDH: F-5′-AAT GGA TTT GGA CGC ATT GGT-3′ and R-5′-TTT GCA CTG GTA CGT GTT GAT-3′.

### Western Blot analysis

The total protein was extracted from MC3T3-E1 cells with M-PER Mammalian Protein Extraction Reagent (Thermo Scientific, USA) including a 10% protease inhibitor cocktail (Roche, Switzerland). The protein concentration was measured by a Pierce^™^ BCA Protein Assay Kit (Thermo Scientific, USA). Equal amounts of protein samples containing loading buffer were subjected to electrophoresis on NuPAGE^™^ Bis–Tris Protein Gels (Invitrogen, USA) for 2 h. Proteins were transferred to polyvinylidene difluoride membranes, and the membranes were blocked in 5% skim milk for 4 h. Then, the PVDF membranes were incubated with primary antibodies specific for Runx2 (1:1000, Cell Signaling Technology, USA), Caspase-3 (1:1000, Cell Signaling Technology, USA), and β-actin (1:1000, Cell Signaling Technology, USA) overnight at 4 °C. The next day, after washing with TBST, the PVDF membranes were incubated with peroxidase-conjugated secondary antibodies (1:5000, ZSGB-BIO, China) for 1 h. The signals were visualized by Super Signal West substrate (Thermo Fisher Scientific, USA). Densitometric analyses of the bands were performed using Tanon Imaging software.

### Flow cytometry

MC3T3-E1 cells were washed with PBS and centrifuged for 5 min at 1000 rpm after digested with 0.125% trypsin solution. Then, cells were resuspended and stained by an Annexin V-FITC Apoptosis Detection Kit (BioVision, USA). Apoptosis rates were analyzed by flow cytometry (BD Bioscience, USA).

### Micro-CT analysis

The femur was scanned by micro-CT (model: vivaCT40; Scanco Medical, Bassersdorf, Switzerland). The sample was scanned at 360°, and 100 µCT slices were acquired from the metaphyseal growth plate. Bone volume (BV), bone volume fraction (BV/TV), connectivity density (Conn. D), trabecular thickness (Tb. Th), trabecular number (Tb. N), and trabecular separation (Tb. Sp) were chosen as the structural parameters in the analysis.

### Masson staining

The fixed femurs were decalcified by decalcifying solution with EDTA (Sigma-Aldrich, Missouri, USA). Then, the samples were embedded in paraffin. Four-micrometer sections were prepared by a rotation microtome (Thermo, Massachusetts, USA). Bone sections were labeled with Masson staining according to the manufacturer’s protocol (Sigma-Aldrich, Missouri, USA).

### Bone metabolism markers

The levels of BALP and CTX-1 in the serum were measured with ELISA kits (R&D System, Inc. MN, USA) following the manufacturer’s instructions. Then, the absorbance was assessed at 450 nm (Pharmacia Biotech, Sweden).

### Statistic analysis

Data were analyzed with SPSS 18.0 software, which was expressed as the mean ± SD. The two-group samples were analyzed by Student’s *t* test, and multiple independent groups were analyzed by one-way ANOVA. *P* < 0.05 was considered to be significant.

### Reporting summary

Further information on experimental design is available in the Nature Research Reporting Summary linked to this paper.

## Supplementary information

Supplementary Figure

Reporting Summary Checklist

## Data Availability

The data of this study are available from the authors upon reasonable request.
