# Supplementary material for: The combined effects of simulated microgravity and X-ray radiation on MC3T3-E1 cells and rat femurs
Source: NPJ Microgravity. 2021 Feb 15;7:3. doi: 10.1038/s41526-021-00131-1 (PMC7884416; doi:10.1038/s41526-021-00131-1)
Supplement: Supplementary file 1 — Supplementary Figure [file 41526_2021_131_MOESM1_ESM.pdf]

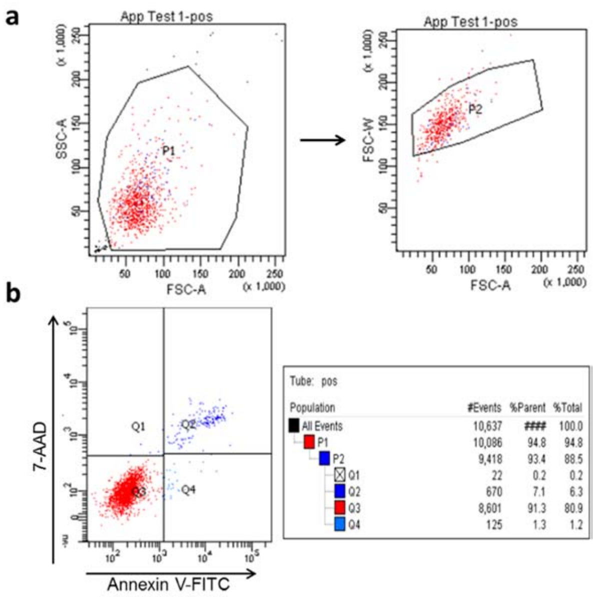

Supplementary Figure 1: FACS sequential gating strategies

(a) P1 is the total MC3T3-E1 cells ($1x10^5$) flow cytometry dot plots, and P2 is the apoptotic analyzed MC3T3-E1 cells flow cytometry dot plots. (b) Apoptotic monitoring of the MC3T3-E1 cells treated with CON, MG，RA and MG+RA was performed by staining with PE-conjugated Annexin V and 7-amino actinomycin D (7-AAD). The percentages of Annexin V positive (+)/7AAD negative (-) cells (Q4 quadrant representing cells in the early stage of apoptosis) and Annexin V positive(+)/7-AAD positive(+) cells (Q2 quadrant representing cells in the late stage of apoptosis) were determined using the flow cytometry (BD Bioscience, USA) with a FAC Scan system.

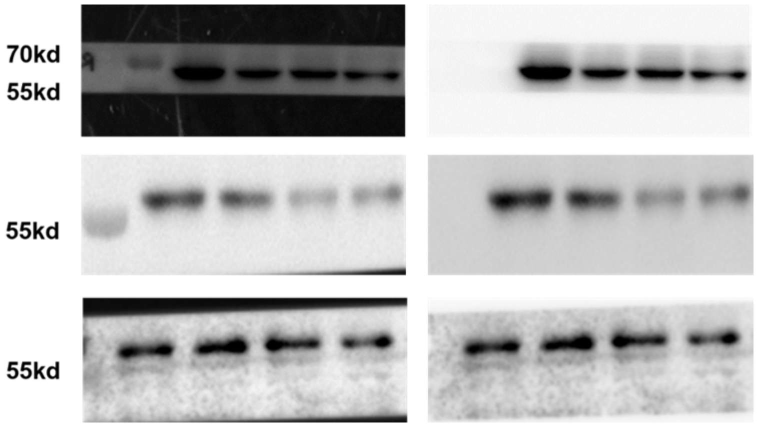

Supplementary Figure 2: Western blot images of osteoblast marker genes (Runx2) in the four groups of MC3T3-E1 cells (n=3).

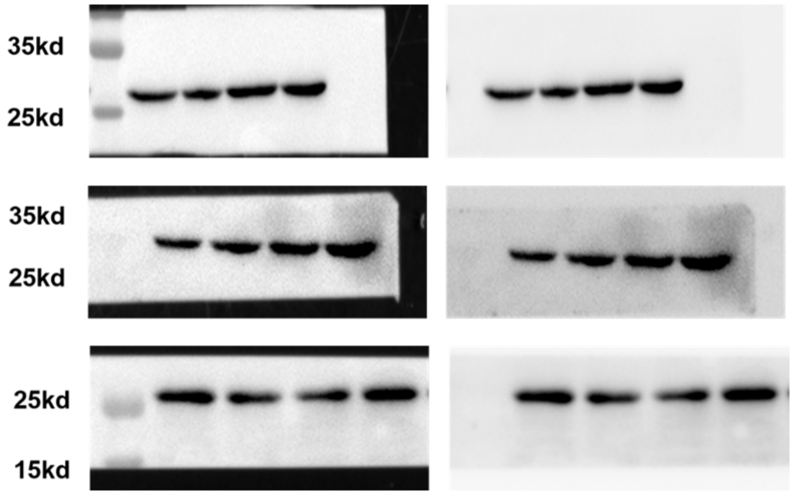

Supplementary Figure 3: Western blot images of osteoblast marker genes (Caspase-3) in the four groups of MC3T3-E1 cells (n=3).
