# Supplementary material for: The combined effects of simulated microgravity and X-ray radiation on MC3T3-E1 cells and rat femurs
Source: NPJ Microgravity. 2021 Feb 15;7:3. doi: 10.1038/s41526-021-00131-1 (PMC7884416; doi:10.1038/s41526-021-00131-1)
Supplement: Supplementary file 2 — Reporting Summary Checklist [file 41526_2021_131_MOESM2_ESM.pdf]

nature research

Corresponding author(s): Shu Zhang

Last updated by author(s): Sep 17, 2020

Reporting Summary

Nature Research wishes to improve the reproducibility of the work that we publish. This form provides structure for consistency and transparency in reporting. For further information on Nature Research policies, see our Editorial Policies and the Editorial Policy Checklist.

Statistics

For all statistical analyses, confirm that the following items are present in the figure legend, table legend, main text, or Methods section.

n/a	Confirmed	
☐	☒	The exact sample size (n) for each experimental group/condition, given as a discrete number and unit of measurement
☐	☒	A statement on whether measurements were taken from distinct samples or whether the same sample was measured repeatedly
☐	☒	The statistical test(s) used AND whether they are one- or two-sided<br>*Only common tests should be described solely by name; describe more complex techniques in the Methods section.*
☐	☒	A description of all covariates tested
☐	☒	A description of any assumptions or corrections, such as tests of normality and adjustment for multiple comparisons
☐	☒	A full description of the statistical parameters including central tendency (e.g. means) or other basic estimates (e.g. regression coefficient) AND variation (e.g. standard deviation) or associated estimates of uncertainty (e.g. confidence intervals)
☐	☒	For null hypothesis testing, the test statistic (e.g. F, t, r) with confidence intervals, effect sizes, degrees of freedom and P value noted<br>*Give P values as exact values whenever suitable.*
☐	☒	For Bayesian analysis, information on the choice of priors and Markov chain Monte Carlo settings
☒	☐	For hierarchical and complex designs, identification of the appropriate level for tests and full reporting of outcomes
☒	☐	Estimates of effect sizes (e.g. Cohen's d, Pearson's r), indicating how they were calculated

Our web collection on statistics for biologists contains articles on many of the points above.

Software and code

Policy information about availability of computer code

Data collection	Microsoft Excel 2010;Tanon-5200 Software:GelCap Version5.22;
Data analysis	IBM SPSS Statistics Version 11.5; GraphPad Prism 8.0.1.244;Image J win32 1.52p;Adobe Photoshop version: 13.0 6.1 Service Pack 1.

For manuscripts utilizing custom algorithms or software that are central to the research but not yet described in published literature, software must be made available to editors and reviewers. We strongly encourage code deposition in a community repository (e.g. GitHub). See the Nature Research guidelines for submitting code & software for further information.

Data

Policy information about availability of data

All manuscripts must include a data availability statement. This statement should provide the following information, where applicable:

- Accession codes, unique identifiers, or web links for publicly available datasets
- A list of figures that have associated raw data
- A description of any restrictions on data availability

All data generated or used in the study are available from the corresponding author by request.

April 2020

Field-specific reporting

Please select the one below that is the best fit for your research. If you are not sure, read the appropriate sections before making your selection.

☒ Life sciences ☐ Behavioural & social sciences ☐ Ecological, evolutionary & environmental sciences

For a reference copy of the document with all sections, see nature.com/documents/nr-reporting-summary-flat.pdf

Life sciences study design

All studies must disclose on these points even when the disclosure is negative.

Sample size	In viivo teat, rats were randomly assigned to 4 groups (n=6): the microgravity (MG) group, administered hindlimb unloading for 4 w; the radiation (RA) group, exposed to 2 Gy X-ray and then maintained for 4 w; the combined (MG+RA) group, exposed to 2 Gy X-ray and then hindlimb unloading for 4 w; and the control (CON) group, which was maintained for 4 w without any treatment.<br>In vitro test, Cells were randomly grouped into 4 groups: the microgravity (MG) group, which was rotated in a 2D clinorotation for 48 h; the radiation (RA) group, which was exposed to 2 Gy X-ray and cultured for 48 h; the combined (MG+RA) group, which was first exposed to 2 Gy X-ray followed by rotation in a 2D clinorotation for 48 h; and the control (CON) group, which was cultured for 48 h without any treatment.<br>However, cell counting (n=4); qRT-PCR and Western blot analysis(n=3); flow cytometry(n=3).
Data exclusions	No data exclusion.
Replication	All test in this study could be replicated and all attempts at replication were successful.
Randomization	The samples and the animals used in this study were assigned to 4 groups at random.
Blinding	The investigators were blinded to group allocation.

Reporting for specific materials, systems and methods

We require information from authors about some types of materials, experimental systems and methods used in many studies. Here, indicate whether each material, system or method listed is relevant to your study. If you are not sure if a list item applies to your research, read the appropriate section before selecting a response.

Materials & experimental systems

n/a	Involved in the study	
☐	☒	Antibodies
☐	☒	Eukaryotic cell lines
☒	☐	Palaeontology and archaeology
☐	☒	Animals and other organisms
☒	☐	Human research participants
☒	☐	Clinical data
☒	☐	Dual use research of concern

Methods

n/a	Involved in the study	
☒	☐	ChIP-seq
☐	☒	Flow cytometry
☒	☐	MRI-based neuroimaging

Antibodies

Antibodies used	1.Runx2 Rabbit mAb:Cell Signaling Technology Inc,Catalog number:#8486S;Lot No:19<br>2.Caspase-3 Polyclonal antibody,Proteintech Inc;Catalog number:19677-1-AP;Clone name:Caspase-3 Rabbit Polyclonal antibody;Lot No:00083623;<br>3.beta-Actin Rabbit mAb:Cell Signaling Technology Inc,Catalog number:#8457S;Lot No:6<br>4.Anti-rabbit IgG HRP linked Antibody:Cell Signaling Technology Inc,Catalog number:#7074S;Lot No:28
Validation	Web:www.cellsignal.com; www.ptgcn.com

Eukaryotic cell lines

Policy information about cell lines

Cell line source(s)	The pre-osteoblast MC3T3-E1 cells were used as the research object in this study.
Authentication	None of the cell lines used were authenticated.
Mycoplasma contamination	The cell lines were not tested for mycoplasma contamination.

Commonly misidentified lines (See ICLAC register)	None of the commonly misidentified lines were used in this study.

Animals and other organisms

Policy information about studies involving animals; ARRIVE guidelines recommended for reporting animal research

Laboratory animals	Seven-week-old male Sprague-Dawley (SD) rats were chosen for this study.
Wild animals	This study did not involve wild animals.
Field-collected samples	To simulate microgravity, rats were suspended by the tail individually, using a strip of adhesive surgical tape attached to a chain hanging on a pulley, and maintained at an angle of 30° with only the forelimbs on the floor for 4 w. The suspended rats could move freely.<br>To simulate radiation, rats were anesthetized and irradiated in the prone position with a single field of 180 kV X-rays to a dose of 2 Gy at a rate of 2 Gy/min (MultiRad225; Faxitron, USA). Cells were placed in the vessel, and then irradiated at a dose of 2 Gy X-rays.<br>The rats were feed at the room temperature. 4w later, the rats were euthanized after anesthesia.
Ethics oversight	All rats were maintained in accordance with the guidelines of the Committees of Animal Ethics and Experimental Safety of the Air Force Medical University. All experimental protocols were approved by the Animal Care Committee of Air Force Medical University.

Note that full information on the approval of the study protocol must also be provided in the manuscript.

Flow Cytometry

Plots

Confirm that:

☒ The axis labels state the marker and fluorochrome used (e.g. CD4-FITC).

☒ The axis scales are clearly visible. Include numbers along axes only for bottom left plot of group (a 'group' is an analysis of identical markers).

☒ All plots are contour plots with outliers or pseudocolor plots.

☒ A numerical value for number of cells or percentage (with statistics) is provided.

Methodology

Sample preparation	1. Wash cells twice with cold PBS and then resuspend cells in 1X Binding Buffer at a concentration of 1 x 10^6 cells/ml.<br>2. Transfer 100 µl of the solution (1 x 10^5 cells) to a 5 ml culture tube.<br>3. Add 5 µl of PE Annexin V and 5 µl 7-AAD.<br>4. Gently vortex the cells and incubate for 15 min at RT (25°C) in the dark.<br>5. Add 400 µl of 1X Binding Buffer to each tube. Analyze by flow cytometry within 1 hr.
Instrument	BD FACSAria II
Software	BD FACS Diva Software version 6.1.3(build 2009 05 13 13 29) Firmware Version 1.6
Cell population abundance	The apoptosis of the treated cells were test only without cell population abundance in this study.
Gating strategy	Data from 10,000 cells per sample was collected, stored and analyzed. Five data parameters were collected simultaneously for each cell: FS; SS; FITC-green fluorescence, PE-orange fluorescence and 7-AAD-red fluorescence. Fluorochrome compensation settings were adjusted using CD4-FITC/CD45-PE/7-AAD staining of whole cells. The two-dimensional log CD45-PE versus SS was used to gate leukemic cells (myeloblasts). The two-dimensional 7-AAD versus log DNA strand breaks-FITC was used to discriminate apoptosis of the gated cell populations.

☒ Tick this box to confirm that a figure exemplifying the gating strategy is provided in the Supplementary Information.
